# Tubular and Glomerular Kidney Effects in Swedish Women with Low Environmental Cadmium Exposure

**DOI:** 10.1289/ehp.8033

**Published:** 2005-07-11

**Authors:** Agneta Åkesson, Thomas Lundh, Marie Vahter, Per Bjellerup, Jonas Lidfeldt, Christina Nerbrand, Göran Samsioe, Ulf Strömberg, Staffan Skerfving

**Affiliations:** 1 Institute of Environmental Medicine, Division of Metals and Health, Karolinska Institutet, Stockholm, Sweden; 2 Department of Occupational and Environmental Medicine, University Hospital, Lund, Sweden; 3 Department of Clinical Chemistry, Karolinska University Hospital, Huddinge, Sweden; 4 Department of Community Health, Malmö University Hospital, Malmö, Sweden; 5 Department of Medicine, and; 6 Department of Gynecology and Obstetrics, University Hospital, Lund, Sweden

**Keywords:** cadmium, diabetes, environmental exposure, glomerular effects, hypertension, kidney, lead, population-based, tubular effects, women

## Abstract

Cadmium is a well-known nephrotoxic agent in food and tobacco, but the exposure level that is critical for kidney effects in the general population is not defined. Within a population-based women’s health survey in southern Sweden (Women’s Health in the Lund Area, WHILA), we investigated cadmium exposure in relation to tubular and glomerular function, from 1999 through early 2000 in 820 women (71% participation rate) 53–64 years of age. Multiple linear regression showed cadmium in blood (median, 0.38 μg/L) and urine (0.52 μg/L; density adjusted = 0.67 μg/g creatinine) to be significantly associated with effects on renal tubules (as indicated by increased levels of human complex-forming protein and *N*-acetyl-β-d-glucosaminidase in urine), after adjusting for age, body mass index, blood lead, diabetes, hypertension, and regular use of nephrotoxic drugs. The associations remained significant even at the low exposure in women who had never smoked. We also found associations with markers of glomerular effects: glomerular filtration rate and creatinine clearance. Significant effects were seen already at a mean urinary cadmium level of 0.6 μg/L (0.8 μg/g creatinine). Cadmium potentiated diabetes-induced effects on kidney. In conclusion, tubular renal effects occurred at lower cadmium levels than previously demonstrated, and more important, glomerular effects were also observed. Although the effects were small, they may represent early signs of adverse effects, affecting large segments of the population. Subjects with diabetes seem to be at increased risk.

Identification of risk factors for chronic renal failure is essential in order to prevent reduction of life quality and life expectancy and to minimize the high costs of treatment. Cadmium is a widespread environmental pollutant known to cause renal damage (Järup et al. 1998). Apart from smoking, the major sources of cadmium exposure in the general population are cereals, vegetables, and shellfish. There is increasing evidence that toxic effects may occur at much lower exposure levels (Alfven et al. 2000; Buchet et al. 1990; Järup et al. 2000; Noonan et al. 2002) than those observed in occupational settings or in severely polluted environments. Still, the attempts to estimate the level of critical exposure for kidney effects have so far displayed large variations. Furthermore, possible effects in populations residing in areas with no particular industrial cadmium emission are undetermined.

Cadmium accumulates in the renal cortex and induces tubular toxicity (Barbier et al. 2005), which is first detected as increased urinary excretion of low-molecular-weight proteins and tubular enzymes. Glomerular dysfunction may also emerge, as demonstrated in heavily exposed subjects (Järup et al. 1995; Kido et al. 1990; Roels et al. 1989). It is not known, however, whether the glomerulus is affected by long-term low-level environmental exposure. Diabetes, an increasing health problem in many areas (King et al. 1998) and one of the leading causes of incident end-stage renal disease (Hostetter 2001), has been suggested to augment the risk of cadmium-induced kidney damage (Buchet et al. 1990). Also, hypertension and intake of nephrotoxic nonsteroid anti-inflammatory drugs (NSAIDs) (Fored et al. 2001) might interact with cadmium. However, these possible interactions need to be confirmed.

The aim of the present investigation was to assess the association between cadmium concentrations in blood and urine and a series of markers of tubular and glomerular function. To minimize dilution of the effects, we focused on women at the age when the accumulation of cadmium in the kidney is at its maximum. Women have increased cadmium accumulation compared with men (Järup et al. 1998; Nishijo et al. 2004b), due to a higher dietary cadmium absorption at low body iron stores (Åkesson et al. 2002; Berglund et al. 1994). In addition, we assessed whether diabetes, hypertension, and the use of NSAIDs increased the risk. The study was conducted in an area without particular industrial cadmium emission.

## Materials and Methods

### Study population.

The Women’s Health in the Lund Area (WHILA) study, a population-based study of all women 50–59 years of age in the community of Lund, southern Sweden (*n* = 10,766), started in December 1995 (Lidfeldt et al. 2001) and was extended in June 1999 to include health aspects of cadmium. This cohort was considered optimal for elucidation of remaining questions about dose–response relationships at low-dose cadmium exposure. The participation rate was 71% (*n* = 820). The exclusion criteria were renal cancer (*n* = 1) and lithium treatment (*n* = 3). Data were collected on various comorbidities, including diabetes and hypertension. Women were classed as having diabetes if they had a positive history, or if they had a non-fasting glucose > 8 mmol/L followed by a positive result in the oral glucose-tolerance test. Women were classed as hypertensive if they had received antihypertensive treatment or had a measured systolic and/or diastolic blood pressure ≥160 and ≥95, respectively (mean of two measurements after 15 min rest in seated position). Lists of medications and data on smoking were obtained, and weight and height were measured. Participants were asked to submit morning first-voided urine and blood samples. We obtained morning spot urine from 813 women and blood samples from 742. All samples were collected during 8 months from June 1999 through January 2000. The ethics committee at Lund University approved the WHILA study, and oral informed consent was obtained.

### Analyses of exposure and kidney function.

Measurements included cadmium in blood as a measure mainly of ongoing exposure (expected to be fairly constant over time) and urine as a measure of body burden (cadmium in urine correlates well with cadmium in the kidney cortex; Järup et al. 1998; Orlowski et al. 1998). To control for possible confounding/effect modification, we also determined lead in blood (Lin et al. 2003). We used the following effect markers: cystatin C in serum (Dharnidharka et al. 2002) for calculation of glomerular filtration rate (GFR), creatinine clearance as markers of glomerular function, and human complex-forming protein (protein HC, α_1_-microglobulin), *N*-acetyl-β-d-glucosaminidase (NAG), and calcium in urine as markers of tubular damage.

We measured cadmium, lead, and calcium using inductively coupled plasma mass spectrometry (Barany et al. 1997). Cystatin C was determined by immunonephelometry (Dade Behring, Marburg, Germany). GFR = 77.24 × (cystatin C)^−1.2623^ (Larsson et al. 2004) and creatinine clearance = [(140 – age) × body weight (kg)]/[0.85 × serum creatinine (μM)] (Harmoinen et al. 2003). Creatinine was measured using a modified kinetic Jaffé method (Roche Diagnostics, Mannheim, Germany). We determined urinary protein HC by Mancini technique and polyclonal antibodies (DAKO A/S, Glostrup, Denmark) (Järup et al. 2000), and urinary NAG with a colorimetric method (Roche, Shionogi & Co. Ltd., Osaka, Japan).

Urinary spot samples need to be adjusted for dilution. Creatinine adjustment is most common, but a comparison of density and creatinine adjusted urinary cadmium indicated that creatinine did not adjust for all dilution-related variation of cadmium in urine. Because creatinine excretion is dependent upon meat intake and muscle mass (Davies et al. 2002; Suwazono et al. 2005), we chose to correct all urinary markers by the mean urinary density (1.015 g/mL) according to [urinary cadmium × (1.015 × 1,000) –1,000]/[(urinary density × 1,000) – 1,000]. However, creatinine-adjusted values are given for comparison.

### Analytical performance.

All the equipment was tested, and possible contamination was below the limit of detection. For cadmium and lead in blood and cadmium and calcium in urine, the limits of detection were 0.12 μg/L, 0.26 μg/L, 0.31 μg/L, and 1.6 mg/L, respectively. For results below the limit of detection (mainly urinary cadmium), the concentration was set as the value factually obtained in the analysis. The imprecision of the method, calculated as the coefficient of variation for duplicate measurements, was 7.4 and 3.1% for cadmium and lead in blood and 8.5 and 6.4% for cadmium and calcium in urine. The analytical accuracy for blood (Seronorm, batch 404107; Nycomed, Oslo, Norway) was as follows (mean ± SD): 0.67 ± 0.08 for cadmium and 29 ± 1.1 for lead (*n* = 21; recommended, 0.67–0.70 and 31–39 μg/L, respectively). The data for certified blood samples from the U.K. National External Quality Assessment Service (*n* = 11) deviated on average by ± 7.9% for target values of 1.8–8.9 μg cadmium/L and ± 6.1% for 52–352 μg lead/L. The results for urine (Seronorm, batch 102021) were 140 ± 8.1 mg/L (*n* = 20; recommended 130 mg/L) for calcium and 0.45 ± 0.07 μg/L (*n* = 20; recommended, 0.35 μg/L) for cadmium. The result for the certified urine samples from Centre de Toxicologie du Quebec Interlaboratory Comparison Program for cadmium was 0.76 ± 0.09 and 3.6 ± 0.22 μg/L (*n* = 11; certified 0.79 and 3.6 μg/L), respectively.

The imprecision was 2.7% for cystatin C (*n* = 6), 16% for protein HC (*n* = 10; limit of detection = 0.7 mg/L), and < 10% for urinary NAG (*n* = 68).

### Statistical analyses.

We used Spearman’s rank correlation analysis to assess univariate associations. The cadmium-associated kidney effect markers were further evaluated in multiple linear regression models, where each kidney effect marker was evaluated in relation to cadmium and confounders/covariates. The dependent effect markers (continuous) were not dichotomized (Ragland 1992). No log-transformation was needed as indicated by residual and goodness-of-fit analyses. We evaluated possible effect modification (interactions) for cadmium and lead. Lowest observed effect levels were assessed for each effect marker for categorized urinary cadmium using Dunnett’s post hoc test, including significant confounders and covariates in the models. All tests were two sided, and statistics were performed using SPSS (version 12.01; SPSS Inc., Chicago, IL, USA).

## Results

The study population characteristics, exposure variables, and kidney effect markers are presented in Table 1. The proportion of subjects with diabetes was slightly higher in the present study compared with those participating in the whole WHILA cohort (6.4%) (Lidfeldt 2003). The proportion of hypertensive subjects was, however, similar (Lidfeldt 2003). Those who had ever smoked had 90% higher cadmium concentrations in blood and 40% higher in urine compared with never-smokers, who had 0.30 μg/L and 0.45 μg/L cadmium in blood and urine, respectively.

The univariate associations between cadmium and kidney effect markers as well as those with possible confounders and effect modifiers are shown in Table 2. Cadmium in both blood and urine was associated with all kidney effect markers except serum creatinine and urinary calcium, which were not included in further analysis. Using cystatin C instead of estimated GFR, and creatinine-adjusted markers in urine instead of density adjusted ones had no major impact on the results.

### Multivariate analyses.

In the multiple linear regression analysis, each of the cadmium-associated kidney effect markers was tested separately, as were cadmium levels in urine and blood (Table 3). We included in the models the covariates age, body mass index (BMI), and blood lead, as well as the possible kidney-effect modifiers diabetes, hypertension, and use of NSAIDs. Never-smokers were analyzed separately. Cadmium in urine was significantly associated with GFR, creatinine clearance, protein HC, and NAG, after controlling for confounders and adjusting for other covariables (Table 3). Similar results were obtained for blood cadmium. In never-smokers, cadmium remained associated with protein HC and NAG and became significantly associated with creatinine clearance (blood cadmium). Lead was significantly associated with GFR and creatinine clearance.

Because there were no associations between cadmium and GFR or creatinine clearance (for urinary cadmium) in never-smokers, we tested whether there was confounding through a non-cadmium-dependent effect of smoking by including pack-years in the multiple regression models. Smoking (pack-years) was not significantly associated with GFR or creatinine clearance (*p* ≥0.1).

We assessed possible interactions between cadmium and blood lead, diabetes (insulin-treated vs. the rest), hypertension, or use of NSAIDs, and between blood lead and diabetes, hypertension, or use of NSAIDs. For NAG, there was an interaction between urinary cadmium and diabetes (insulin-treated vs. other diabetics and nondiabetics; regression coefficients (β): diabetics = 2.3, nondiabetics = 0.8; *R*^2^ = 0.10; *p* = 0.042) (Table 3). For protein HC, there was a close to significant interaction between urinary cadmium and diabetes (β: diabetics = 4.0, nondiabetics = 1.3; *R*^2^ = 0.09; *p* = 0.07), which became significant in never-smokers (β: diabetics = 27, nondiabetics = 1.9; *R*^2^ = 0.18; *p* < 0.001). Similar interactions were observed between blood cadmium and diabetes. Hypertension, NSAID use, and blood lead showed no significant interactions with cadmium exposure. However, there was an interaction between blood lead and diabetes for GFR in never-smokers (*p* = 0.005).

### Lowest observed effect level.

**Figure 1:**
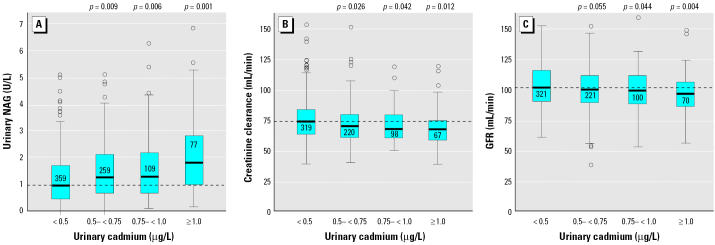
Associations (crude) between urinary NAG (*A*), creatinine clearance (*B*), and GFR (*C*) and urinary cadmium (categorized) in a population-based study from 1999 through early 2000 on 816 women in southern Sweden. Boxes indicate 25th, 50th (solid line), and 75th percentiles, and whiskers indicate minimum and maximum, excluding outliers (circles; a few, not shown in the figure but included in all the calculations). Numbers inside boxes indicate the number of samples. The dashed line indicates the median in the lowest urinary cadmium exposure category. *p*-Values for differences between the lowest exposure group and the following groups are indicated (Dunnett’s test including the significant confounders and covariates according to Table 3).

Protein HC, NAG (diabetics excluded) (Figure 1A), and creatinine clearance (Figure 1B), after adjustment for blood lead, differed significantly between the group with lowest exposure level (urinary cadmium < 0.5 μg/L; mean, 0.36 μg cadmium/L = 0.48 μg cadmium/g creatinine) and that with the next lowest exposure level (0.50–0.75 μg/L; mean, 0.61 μg cadmium/L = 0.79 μg cadmium/g creatinine). For GFR, the group with the next highest exposure level [urinary cadmium, 0.75–1 μg cadmium/L; mean, 0.86 μg cadmium/L = 1.0 μg cadmium/g creatinine; adjusted for age, BMI, and blood lead (each categorized into four groups) and for NSAID use (into 0 or 1); Figure 1C] differed from the lowest level. For blood cadmium, associations were present in the exposure category 0.5–1 μg/L (mean, 0.69 μg/L) for protein HC (*p* = 0.036) and NAG (*p* = 0.024). For GFR, an association was seen only at blood cadmium > 1 μg/L (mean, 1.8 μg/L; *p* < 0.001) after adjustment for significant covariates.

## Discussion

This population-based study of upper-middle-age women, representative of the general population of southern Sweden, showed clear associations between cadmium and the renal tubular-effect markers protein HC and NAG, even at the low levels of cadmium found in never-smokers. Cadmium potentiated the diabetes-induced effects on the kidney. There was also a clear association between cadmium and GFR or creatinine clearance.

This study has several methodologic advantages, including the large sample size and high participation rate, individual exposure assessment with high analytical accuracy, and inclusion of several different outcomes of renal effects. Despite the low cadmium concentrations, we had a high analytical accuracy. Any imprecision would have caused a bias toward the null.

The study population differed somewhat from the total WHILA population and Sweden (4–7%) (Lidfeldt 2003). Hence, there was a slight overrepresentation of diabetics, which may cause an overestimate of cadmium effects. However, because we controlled for diabetes in the statistical models, this is not a problem. Overcontrol and collinearity may occur in a statistical analysis such as that performed in this study. Smoking is then an obvious problem, which we handled by separate analysis in never-smokers. Lead and BMI were included, which means a risk of some overcontrol.

Another problem, common in the interpretation of data from cross-sectional studies, is that the exposure is measured at the same time as the effects, which may not be the etiologically relevant period. This may be problematic for blood cadmium, because it largely reflects recent exposure, but not for urinary cadmium, which is a good estimate of the integrated low-level exposure over decades (Järup et al. 1998). It is known that kidney deterioration, due to both aging and high cadmium exposure, increases the excretion of cadmium in urine, resulting in lower kidney cadmium and eventually lower urinary cadmium. However, the present participants were below the age when the kidney cadmium starts to decrease, and the exposure was relatively low.

The present cadmium concentrations are comparable with, or slightly higher than, those in other recent studies from Sweden (Åkesson et al. 2002; Järup et al. 2000; Olsson et al. 2002) and the United States [Centers for Disease Control and Prevention (CDC) 2003; Noonan et al. 2002; Paschal et al. 2000] but lower than those in more contaminated areas of Europe (Buchet et al. 1990; Hotz et al. 1999) and much lower than in certain areas in Japan (Suwazono et al. 2000; Yamanaka et al. 1998). Despite the present low cadmium levels, there were clear effects on the kidney. The associations between cadmium and biomarkers of several different renal effects support causality. It is unlikely that they are merely a result of parallel phenomena, impaired tubular reabsorption (protein HC), or increased general turnover of tubular cells (NAG). The associations with blood cadmium also preclude such an interpretation. Because smoking is a major source of cadmium exposure (Järup et al. 1998), the possibility of confounding through a non-cadmium-dependent effect of smoking must be considered. However, because we found cadmium-associated effects on NAG and protein HC even in never-smokers, and there was no effect of smoking on creatinine clearance or GFR, this is unlikely.

The lead levels were low. The association between blood lead and GFR and creatinine clearance may indicate either an effect on GFR at low lead exposure (Lin et al. 2003) or reverse causality. In the case of cadmium, reverse causality seems highly unlikely. Even though data may imply that a decrease in GFR causes increased blood cadmium concentrations, the inverse associations between the glomerular effect markers and cadmium in urine rather indicate that reduced GFR does not reduce the clearance of cadmium. In addition, lead is bound to high-molecular-weight plasma albumin, and cadmium to metallothionein, a small polypeptide that is easily filtered through the glomerulus.

The lowest observed effect level, defined as the mean urinary cadmium in the exposure category that displayed significantly different levels of effect markers compared with the lowest urinary cadmium category, was 0.6 μg cadmium/L (0.8 μg/g creatinine), corresponding to approximately 20 μg cadmium/g kidney cortex. The lowest observed effect level is lower than in previous studies that observed effects at low-level cadmium exposure (Buchet et al. 1990; Järup et al. 2000; Noonan et al. 2002). This is probably due to homogeneity of the population, absence of healthy worker effects (Järup et al. 2000), and good precision in analyses of the exposure and effect markers.

More important, we found a similar lowest observed effect level for creatinine clearance as for the tubular markers. Although usually considered an index of glomerular function, the creatinine clearance may partly reflect a proximal tubular dysfunction, because creatinine is not only filtrated but also secreted in the tubuli (Wuyts et al. 2003). On the other hand, the cadmium-associated increase in GFR, occurring in the next highest cadmium stratum (0.86 μg/L urine = 1.0 μg/g creatinine), clearly indicates an effect on the glomerular function. An observation in this contex that supports an effect of cadmium on GFR is the reported ecologic association between end-stage renal disease and distance to cadmium-emitting industrial plants (Hellström et al. 2001).

Cadmium has been suggested to cause hypertension, but no such effect was seen here, in agreement with other studies (Staessen et al. 2000). Also, we did not observe any synergism between cadmium and hypertension on the kidney effects. However, there might be a dilution of the group by cases with mild hypertension. Further, it has been reported, both from experimental and epidemiologic studies, that cadmium increases the risk of type II diabetes (Han et al. 2003; Schwartz et al. 2003), which was not supported by the present study. As expected, diabetes affected the kidney function, although only in insulin-dependent women, of whom about half had type II diabetes. More important, we found an interaction between cadmium and diabetes, as suggested in previous studies (Buchet et al. 1990). Hence, the lowest observed effect level is expected to be lower in diabetics but could not be evaluated because of too few cases. The incidence of diabetes is increasing (King et al. 1998), and because diabetes is the leading cause of end-stage renal disease (Hostetter 2001), this has important public health implications. The incidence of renal replacement therapy in Sweden is 125 per million, with an estimated increased prevalence of 5% per year (Swedish National Board of Health and Welfare 2003).

The nephrotoxic effects in the present study appear small in a clinical context, and only a few percent of the variances were explained by cadmium. However, the increase in the effect markers indicates renal toxicity, which should be considered an early sign of severe health effects (Nishijo et al. 2004a). Because it concerns a large segment of the population worldwide, the results are of public health concern. Although the cadmium-induced kidney effects in several studies have been associated with decreasing GFR (Järup et al. 1995; Kido et al. 1990; Roels et al. 1989), a positive aspect is that progression of the very early effect may not always occur when the exposure is substantially decreased (Hotz et al. 1999). It should, however, be emphasized that in areas with exposure to cadmium mainly through diet, the long half-time of cadmium in the soil will hamper a decrease of the exposure. Thus, far-reaching mitigation will be needed in addition to actions against smoking.

## Figures and Tables

**Table 1:** Participant characteristics and data on exposure and kidney effect markers in a population-based study from 1999 through early 2000 on 816 women in southern Sweden.

Variable (unit)	Median (5–95% percentiles)	No. of samples
Population characteristic		
Age (years)	58 (54–63)	816
BMI (kg/m^2^)	26.2 (20.3–33.9)	
Smokers: never/former/current (%)	54/25/21^a^	
Diabetes: all/insulin dependent (%)	10/1.7^a^	
Hypertension: all/drug treated (%)	31/18^a^	
Regular use of NSAIDs (%)	6^a^	
Exposure variables		
Blood cadmium (μg/L)	0.38 (0.16–1.8)	725
Urinary cadmium (μg/L)^b^	0.52 (0.24–1.3)	807
Urinary cadmium (μg/g creatinine)	0.67 (0.31–1.6)	
Blood lead (μg/L)	22 (11–46)	726
Kidney effect markers		
Serum cystatin C (mg/L)	0.81 (0.65–1.0)	721
GFR (mL/min)^c^	101 (74–133)	
Serum creatinine (μmol/L)	92 (73–116)	713
Creatinine clearance (mL/min)^d^	72 (51–105)	
Urinary protein HC (μg/L)^b^	2.4 (0.98–7.9)	806
Urinary protein HC (mg/g creatinine)	3.1 (0.13–1.2)	
Urinary NAG (U/L)^b^	1.2 (0.22–3.6)	806
Urinary NAG (U/g creatinine)	1.4 (1.1–11)	
Urinary calcium (mg/L)^b^	135 (56–267)	809
Urinary calcium (mg/g creatinine)	170 (62–366)	

BMI, body mass index.

a Data are presented as percent.

b Adjusted to mean density 1.015 g/mL.

c Calculated: 77.24 × (serum cystatin C)^−1.2623^.

d Calculated: [(140 – age) × body weight (kg)]/[0.85 × serum creatinine (μM)]. Mean urinary creatinine = 0.85 g/L; conversion factors: cadmium: 1 μg = 8.89 nmol; 1.0 μg/g creatinine ≈ 1.0 nmol/mmol creatinine; lead: 1 μg = 4.83 nmol.

**Table 2:** Associations between exposure and effect markers (Spearman’s rank correlation coefficients).

	Age	BMI	Pack-years	Blood cadmium	Urinary cadmium	Blood lead	GFR	Serum creatinine	Creatinine clearance	Urinary protein HC
Blood cadmium	−0.01	−0.14#	0.56#							
Urinary cadmium	−0.02	−0.15#	0.42#	0.57#						
Blood lead	−0.03	−0.08*	0.18#	0.20#	0.15#					
GFR	−0.28#	−0.27#	−0.05	−0.13#	−0.12#	−0.11*				
Serum creatinine	0.12*	−0.08*	0.00	0.02	0.05	0.13#	−0.38#			
Creatinine clearance	NR	NR	−0.02	−0.08*	−0.13#	−0.13#	0.11#	−0.62#		
Urinary protein HC	0.05	−0.18#	0.08*	0.15#	0.18#	−0.01	−0.05	−0.02	−0.11*	
Urinary NAG	0.06	−0.03	0.12#	0.13#	0.23#	0.02	−0.13#	0.09*	−0.09*	0.21#
Urinary calcium	−0.03	−0.04	−0.03	0.01	−0.02	0.12#	0.16#	−0.15#	0.06	−0.04

Abbreviations: BMI, body mass index; NR, not relevant, as included in the calculation of creatinine clearance.

* *p* ≤0.05.

# *p* ≤0.001.

**Table 3:** Associations between markers of cadmium exposure and effects in a population-based study on 816 Swedish women, allowing for other risk factors, performed in all subjects and never-smokers separately.

		All			Never-smokers		
Dependent variable	Independent variable	β	95% CI	*R*^2^	β	95% CI	*R*^2^
GFR (mL/min)	Urinary cadmium (μg/L)	−7.9	−11 to −4.3	0.15	−5.0	−11 to 0.9	0.16
	Age (year)	−1.5	−1.9 to −1.0		−1.3	−1.9 to −0.7	
	BMI (kg/m^2^)	−1.0	−1.3 to −0.7		−1.1	−1.5 to −0.7	
	Blood lead (μg/L)	−0.20	−0.32 to −0.09		−0.26	−0.43 to −0.09	
	Diabetes^a^	NS			−25	−46 to −5.0	
	Hypertension^b^	NS			NS		
	NSAIDs^c^	−6.8	−12 to −1.2		NS		
	Blood cadmium	−4.2	−6.6 to −1.9	0.15	−6.0	−15 to 3.0	0.16
	Age (year)	−1.5	−1.9 to −1.0		−1.2	−1.8 to −0.7	
	BMI (kg/m^2^)	−1.0	−1.3 to −0.7		−1.1	−1.6 to −0.7	
	Blood lead (μg/L)	−0.2	−0.3 to −0.07		−0.2*	−0.4 to −0.07	
	Diabetes	NS			−25	−45 to −4.8	
	Hypertension	NS			NS		
	NSAIDs	−6.1	−12 to −0.5		NS		
Creatinine clearance (mL/min)	Urinary cadmium (μg/L)	−4.3	−8.0 to −0.7	0.03	−3.5	−9.9 to 2.8	0.05
	Blood lead (μg/L)	−0.18	−0.30 to −0.06		−0.3	−0.5 to −0.1	
	Diabetes	NS			−29	−51 to −8.0	
	Hypertension	NS			NS		
	NSAIDs	NS			NS		
	Blood cadmium	−1.6	−4.0 to 0.7	0.03	−9.8	−19 to −0.5	0.06
	Blood lead (μg/L)	−0.18	−0.30 to −0.07		−0.3	−0.5 to −0.1	
	Diabetes	NS			−29	−50 to −8.0	
	Hypertension	3.0	0.3 to 5.9		NS		
	NSAIDs	NS			NS		
Urinary protein HC (μg/L)	Urinary cadmium (μg/L)	1.4	0.9 to 1.8	0.09	2.1*	1.3 to 2.8	0.13
	Age (year)	NS			NS		
	BMI (kg/m^2^)	−0.06	−0.10 to −0.02		−0.08	−0.13 to −0.02	
	Blood lead (μg/L)	NS			NS		
	Diabetes	3.5	2.2 to 4.9		5.1	3.1 to 7.1	
	Hypertension	NS			0.57	0.05 to 1.1	
	NSAIDs	NS			NS		
	Blood cadmium	0.5	0.2 to 0.8	0.06	1.7*	0.5 to 3.0	0.09
	Age (year)	NS			NS		
	BMI (kg/m^2^)	−0.07	−0.11 to −0.02		−0.08	−0.14 to −0.02	
	Blood lead (μg/L)	NS			NS		
	Diabetes	3.5	2.2 to 4.8		5.8	3.4 to 8.1	
	Hypertension	NS			NS		
	NSAIDs	NS			NS		
Urinary NAG (U/L)	Urinary cadmium (μg/L)	0.9*	0.6 to 1.1	0.09	0.8	0.4 to 1.2	0.10
	Age (year)	NS			NS		
	BMI (kg/m^2^)	NS			NS		
	Blood lead (μg/L)	NS			NS		
	Diabetes	1.5	0.9 to 2.2		3.0	1.9 to 4.1	
	Hypertension	NS			NS		
	NSAIDs	NS			NS		
	Blood cadmium	0.4	0.2 to 0.5	0.05	0.5	−0.05 to 1.1	0.06
	Age (year)	NS			NS		
	BMI (kg/m^2^)	NS			NS		
	Blood lead (μg/L)	NS			NS		
	Diabetes	1.5	0.8 to 2.1		2.9	1.7 to 4.0	
	Hypertension	NS			NS		
	NSAIDs	NS			NS		

Abbreviations: β, regression coefficient; 95% CI, 95% confidence interval; adjusted *R*^2^, explained variance; NS, not significant.

a Insulin treated vs. all others, yes = 1.

b Hypertension, yes = 1.

c NSAIDs, yes = 1.

* Significant interaction with diabetes (described in text).
